# *Heligmosomoides polygyrus* Venom Allergen-like Protein-4 (HpVAL-4) is a sterol binding protein

**DOI:** 10.1016/j.ijpara.2018.01.002

**Published:** 2018-04

**Authors:** Oluwatoyin A. Asojo, Rabih Darwiche, Selam Gebremedhin, Geert Smant, Jose L. Lozano-Torres, Claire Drurey, Jeroen Pollet, Rick M. Maizels, Roger Schneiter, Ruud H.P. Wilbers

**Affiliations:** a National School of Tropical Medicine, Baylor College of Medicine, Houston, TX 77030, USA; b Division of Biochemistry, Department of Biology, University of Fribourg, Chemin du Musée 10, CH 1700 Fribourg, Switzerland; c Laboratory of Nematology, Wageningen University, Droevendaalsesteeg 1, 6708 PB Wageningen, The Netherlands; d Wellcome Centre for Molecular Parasitology, Institute for Infection, Immunity and Inflammation, University of Glasgow, Sir Graeme Davies Building, 120 University Place, Glasgow G12 8TA, UK

**Keywords:** Sperm coating protein (SCP), Testis specific proteins (Tpx), Pathogenesis related-1 (PR-1), Cysteine-rich secretory protein (CRISP), Venom antigen 5, Excretory–secretory products, Sterol binding

## Abstract

*Heligmosomoides polygyrus bakeri* is a model parasitic hookworm used to study animal and human helminth diseases. During infection, the parasite releases excretory/secretory products that modulate the immune system of the host. The most abundant protein family in excretory/secretory products comprises the venom allergen-like proteins (VALs), which are members of the SCP/TAPS (sperm-coating protein/Tpx/antigen 5/pathogenesis related-1/Sc7) superfamily. There are >30 secreted *Heligmosomoides polygyrus* VAL proteins (HpVALs) and these proteins are characterised by having either one or two 15 kDa CAP (cysteine-rich secretory protein (CRISP)/antigen 5/pathogenesis related-1) domains. The first known HpVAL structure, HpVAL-4, refined to 1.9 Å is reported. HpVAL-4 was produced as a homogeneously glycosylated protein in leaves of *Nicotiana benthamiana* infiltrated with recombinant plasmids, making this plant expression platform amenable for the production of biological products. The overall topology of HpVAL-4 is a three layered αβα sandwich between a short N-terminal loop and a C-terminal cysteine rich extension. The C-terminal cysteine rich extension has two strands stabilized by two disulfide bonds and superposes well with the previously reported extension from the human hookworm *Necator americanus Ancylostoma* secreted protein-2 (Na-ASP-2). The N-terminal loop is connected to alpha helix 2 via a disulfide bond previously observed in Na-ASP-2. HpVAL-4 has a central cavity that is more similar to the N-terminal CAP domain of the two CAP Na-ASP-1 from *Necator americanus*. Unlike Na-ASP-2, mammalian CRISP, and the C-terminal CAP domain of Na-ASP-1, the large central cavity of HpVAL-4 lacks the two histidines required to coordinate divalent cations. HpVAL-4 has both palmitate-binding and sterol-binding cavities and is able to complement the in vivo sterol export phenotype of yeast mutants lacking their endogenous CAP proteins. More studies are required to determine endogenous binding partners of HpVAL-4 and unravel the possible impact of sterol binding on immune-modulatory functions.

## Highlights

• *Heligmosomoides polygyrus* Venom Allergen-like Protein-4 (HpVAL-4) was produced in plants as a glycosylated protein.
• The crystal structure of HpVAL-4 was solved and reveals three distinct cavities.
• These cavities are the central cavity; the sterol-binding caveolin-binding motif (CBM); and the palmitate-binding cavity.
• The central cavity of Hp-VAL-4 lacks the characteristic histidines that coordinate divalent cations.
• Hp-VAL-4 binds sterol in vivo and in vitro.

## Graphical abstract

**Figure:**
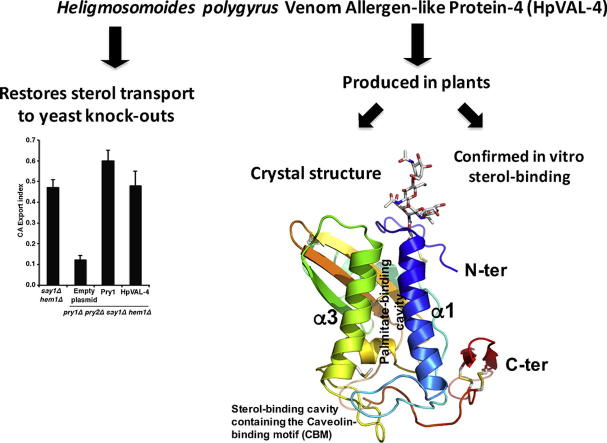

## 1 Introduction

*Heligmosomoides polygyrus bakeri* is a rodent intestinal nematode that is closely related to ruminant and human hookworm parasites. *Heligmosomoides polygyrus* is able to survive long-term in the murine host, and is in widespread use as a model for chronic nematode infections (Behnke, 1987, Robinson et al., 1989). More importantly, *H. polygyrus* has been used as a model to study immunological processes involved in chronic parasitic nematode infections and to characterise host-parasite relationships for over three decades (Behnke, 1987, Robinson et al., 1989, Behnke et al., 2009, Reynolds et al., 2012, Harris et al., 2014). *Heligmosomoides polygyrus* is retained in the intestine and elicits strong systemic Th2 cell responses (Svetic et al., 1993, Wahid et al., 1994). It was also determined that *H. polygyrus* infection drives expansion of regulatory T cells which are able to suppress allergic airway inflammation in mouse models of allergic inflammation (Finney et al., 2007, Rausch et al., 2008, Smith et al., 2016).

*H. polygyrus* secretes immune-modulatory factors and effectors that may account for both immune evasion and suppression evident during infection. Many of the immunomodulatory molecules are released as parasite excretory/secretory (ES) products (Hewitson et al., 2009, Johnston et al., 2009, Ferreira et al., 2013). Passive immunisation with antisera to these ES products confers 50–75% protection against challenge infection (Hewitson et al., 2009). Due to their immunomodulatory properties, ES products from *H. polygyrus* have been explored as putative therapeutic agents for autoimmune and allergic diseases (Harris et al., 2014). Similarly, ES products have been studied as candidate vaccine antigens for hookworm infection and schistosomiasis (Verjovski-Almeida et al., 2003, Bethony et al., 2005, Fujiwara et al., 2005).

Proteomic analysis of ES products of adult *H. polygyrus* revealed that the most abundant proteins are members of the venom allergen-like protein (VAL) family, and among the major ones is *H. polygyrus* VAL-4 (HpVAL-4) (Hewitson et al., 2011b). It was previously shown that homologues of VALs known as *Ancylostoma* secreted proteins (ASPs) are the predominant molecules secreted by canine and human hookworms (Cantacessi et al., 2010, Wang et al., 2010b). The immunomodulatory functions of ASPs are well characterised and some are being explored as vaccines and adjuvants (Fujiwara et al., 2005, He et al., 2009, Gonzalez-Hernandez et al., 2016). VALs are members of the superfamily that is known as CAP (cysteine-rich secretory protein/antigen 5/pathogenesis related-1) or SCP/TAPS (Sperm-coating protein/Tpx/antigen 5/pathogenesis related-1/Sc7). The CAP domain (Pfam PF00188) has been implicated in conditions requiring cellular defense or proliferation including plant responses to pathogens and human brain tumour growth (Hawdon et al., 1999, Ding et al., 2000, Gao et al., 2001, Zhan et al., 2003, Gibbs et al., 2008). In the case of parasite-encoded CAP proteins, many are considered to fulfil immune modulatory functions to sustain survival in the host, and for the same reason are attractive targets for vaccine-induced anti-parasite immunity (Cantacessi et al., 2009).

There are over 30 secreted HpVALs which dominate the immune response in *H. polygyrus* infected mice (Hewitson et al., 2011b). As observed in human and canine hookworms, HpVALs are characterised by having either a single or double 15–25 kDa CAP domain. In infected mice, the antibody response primarily targets four HpVALs, the double-domain HpVAL-1, -2 and -3, and the single domain HpVAL-4. Following secondary infection of mice, antibodies are directed at the same four HpVALs as well as another single domain family member, HpVAL-7. Both HpVAL-1 and HpVAL-2 bear an immunodominant *O*-linked glycan which is exposed on the parasite surface (Hewitson et al., 2011a). Interestingly, each member of the HpVAL family has a distinct pattern of expression during the parasite life cycle with HpVAL-4 being expressed at high levels by all mammalian stages from day 3 and day 5p.i, L3s, L4s, and adult worms (Hewitson et al., 2013)

Several structures of proteins having a single CAP domain and one structure of a hookworm ASP with two CAP domains have been reported (Fernandez et al., 1997, Serrano et al., 2004, Asojo et al., 2005, Guo et al., 2005, Shikamoto et al., 2005, Wang et al., 2005, Gibbs et al., 2008, Asojo, 2011, Xu et al., 2012, Borloo et al., 2013). Each CAP domain has a large central cavity (Serrano et al., 2004, Asojo et al., 2005, Asojo et al., 2011, Gibbs et al., 2008, Suzuki et al., 2008, Wang et al., 2010a, van Galen et al., 2012, Xu et al., 2012, Mason et al., 2014, Darwiche et al., 2016, Baroni et al., 2017). The function of the cavity is unknown but in some SCP/TAPs proteins the cavity contains two histidine residues that have been shown to bind divalent cations including Zn^2+^ and Mg^2+^ (Gibbs and O'Bryan, 2007, Gibbs et al., 2008). The ability to bind Zn^2+^ is critical for heparin-sulphate dependent inflammatory mechanisms of the cobra SCP/TAPs protein natrin (Wang et al., 2010a).

The central CAP domain has a conserved alpha–beta–alpha sandwich topology with variations in the lengths of their strands and helices as well as the lengths, orientations, and locations of loops (Asojo, 2011). These long flexible loops make up as much as 50% of the overall structure of the CAP domain, making it difficult to accurately predict or model their structures (Asojo et al., 2005, Asojo, 2011, Darwiche et al., 2016, Baroni et al., 2017). Interestingly, one of the flexible loop regions of the CAP domain was identified as the sterol-binding caveolin-binding motif (CBM) of yeast CAP proteins required for in vivo cholesterol transport (Choudhary et al., 2014, Darwiche et al., 2016). Two additional lipid-binding regions have been identified in CAP proteins, and all three lipid-binding regions are unique and unconnected in all reported monomer structures of CAP domains (Fernandez et al., 1997, Hawdon et al., 1999, Ding et al., 2000, Gao et al., 2001, Guo et al., 2005, Zhan et al., 2003, Serrano et al., 2004, Asojo et al., 2005, Shikamoto et al., 2005, Wang et al., 2005, Gibbs et al., 2008, Asojo, 2011, Xu et al., 2012, Borloo et al., 2013). The second lipid-binding region is a large hydrophobic cavity between two helices that was identified in horsefly tablysin-15. Tablysin-15 binds leukotrienes in this cavity and functions as an anti-inflammatory scavenger of eicosanoids (Xu et al., 2012). In addition, the yeast CAP protein Pry1 is structurally able to accommodate lipids such as palmitate, and it binds and exports fatty acids similarly to tablysin-15 (Darwiche et al., 2016). The third lipid-binding motif has only been reported on the surface of human Golgi-associated PR-1 protein (GLIPR2) and facilitates the binding of up to three phosphatidylinositol molecules (Van Galen et al., 2010, van Galen et al., 2012). These structural and functional insights into the SCP/TAPS proteins from many different organisms prompted us to analyse a predominant homologue secreted into *H. polygyrus* ES products, HpVAL-4, and we present here the X-ray structure and functional role of HpVAL-4 in cholesterol transport.

## 2 Materials and methods

### 2.1 Plant-based expression of HpVAL-4

The complete sequence encoding mature HpVAL-4 was codon optimised in–house and synthetically constructed at GeneArt. This sequence was cloned into a pHYG expression vector and was preceded by the *Arabidopsis thaliana* chitinase signal peptide (cSP). The HpVAL-4 expression vector was used to transform *Agrobacterium tumefaciens* (strain MOG101) and used for agro-infiltration*.* To enhance expression, the plasmid vector pBIN61 containing the silencing inhibitor p19 from tomato bushy stunt virus was co-infiltrated. HpVAL-4 and p19 *Agrobacterium tumefaciens* clones were grown in Lennox broth (10 g/L of peptone140, 5 g/L of yeast extract, 10 g/L of NaCl pH 7.0) containing 50 μg/ml of kanamycin and 20 μM acetosyringone for 16 h at 28 °C/250 rpm. For agro-infiltration of HpVAL-4 and p19, MMA infiltration medium (20 g/L of sucrose, 5 g/L of Murashige and Skoog basal salt mixture, 1.95 g/L of (*N*-morpholino)ethanesulfonic acid pH5.6) containing 200 μM acetosyringone was used to suspend the bacterial cultures to a final O.D. of 0.5 per culture. A 1 ml needleless syringe was used to infiltrate the *Agrobacterium* suspension into the youngest fully expanded leaves of 5–6 weeks old *Nicotiana benthamiana* plants at the abaxial side. *Nicotiana benthamiana* plants were maintained in a controlled greenhouse compartment (UNIFARM, Wageningen, Netherlands) and infiltrated leaves were harvested at 5–6 days post infiltration.

### 2.2 Purification of HpVAL-4

HpVAL-4 was purified from the leaf extracellular space (apoplast) as described previously (Wilbers et al., 2017). Briefly, the infiltrated leaves were submerged in ice-cold extraction buffer (20 mM sodium citrate pH 3.6, 100 mM NaCl and 0.1% v/v Tween-20). The submerged leaves were vacuum infiltrated and the apoplast fluid was retrieved by centrifugation for 10 min at 2000*g*. The apoplast fluid was clarified by centrifugation for 5 min at 16,000 *g*. HpVAL-4 was then purified from the apoplast fluid using HS POROS 50 strong cation exchange (CEX) resin (Applied Biosystems, USA). Prior to purification the apoplast fluid was passed over a G25 Sephadex column with CEX binding buffer (20 mM sodium citrate buffer pH 3.6, 100 mM NaCl). HpVAL-4 bound to CEX resin was eluted with 20 mM Tris–HCl buffer pH 9.0 containing 2 M NaCl. The purification was performed on an ÄKTA Prime Chromatography System (GE Healthcare, USA) using a constant flow rate of 10 mL/min for binding and washing, and 2 mL/min for elution. Eluted HpVAL-4 was dialyzed overnight in PBS. Recombinant HpVAL-4 was separated under reduced conditions by SDS–PAGE on a 12% Bis-Tris gel (Invitrogen, USA) and subsequently stained with Coomassie brilliant blue staining.

### 2.3 Analysis of N-glycan composition

For N-glycan analysis, 1–2 μg of purified HpVAL-4 was reduced and denatured for 10 min at 95 °C in PBS containing 1.3% (w/v) SDS and 0.1% (v/v) β-mercaptoethanol. SDS was neutralised by adding 2% (v/v) NP-40 prior to overnight digestion at 37 °C with trypsin (Sigma–Aldrich, USA) immobilised to *N*-hydroxysuccinimide-activated Sepharose (GE Healthcare). Trypsin beads were removed from the digestion mix by centrifugation and the pH of the mix was adjusted to 5 using 1 M sodium acetate. PNGase A (0.5 mU; Roche, Switzerland) was used to release N-glycans from HpVAL-4 while incubating overnight at 37 °C. The incubation mixture was applied to C18 Bakerbond™ SPE cartridges (JT Baker, USA) and the N-glycans were extracted from the flow-through on Extract Clean™ Carbo SPE columns. Eluted N-glycans were labelled with anthranilic acid (Sigma–Aldrich) and desalted by hydrophilic interaction chromatography on Biogel P10 (BioRad). Samples in 75% acetonitrile were mixed with 1 μl of matrix solution (20 mg/ml of 2,5-dihydroxybenzoic acid in 50% acetonitrile, 0.1% (v/v) trifluoroacetic) and were dried under a stream of warm air. Matrix-assisted laser desorption/ionisation (MALDI) time-of-flight mass spectra (MS) were obtained using an Ultraflex II mass spectrometer (Bruker Daltonics, USA).

### 2.4 Crystallisation

HpVAL-4 (10 mg/ml) in PBS was screened for crystallisation conditions with commercial screens from Qiagen (Germany), Hampton Research (USA) and Microlytics (USA). Crystals were obtained from multiple conditions with polyethylene glycol, and the best diffracting crystals were obtained at 298 K by vapour diffusion in sitting drops by mixing 1.5 μl of protein solution with an equal volume of the reservoir solution containing 0.1 M sodium acetate trihydrate at pH 4.5, 22.5% (w/v) polyethylene glycol 0.3–8 kD. Since the crystals grew in solutions that contained adequate cryo-protectant, all crystals were flash-cooled directly in a stream of N_2_ gas at 113 K prior to collecting diffraction data.

### 2.5 Data collection and structure determination

X-ray diffraction data were collected at the Baylor College of Medicine, USA, core facility using a Rigaku HTC detector. The X-ray source was a Rigaku FR-E + SuperBright microfocus rotating anode generator with VariMax HF optics. A data set was collected from a single crystal with a crystal-to-detector distance of 105 mm and exposure times of 120 s for 0.5° oscillations, using the Crystal Clear (d∗trek) package (Pflugrath, 1999). Data was processed using MosFLM (Leslie, 2006). The crystal belonged to the triclinic space group *P*_1_ with cell constants *a* = 49.5596 Å *b* = 61.5645 Å *c* = 74.8188 Å, *α* = 111.595° *β* = 90.14° *γ* = 113.475°.

Similar to previous CAP structures, HpVAL-4 structure was solved after several attempts at molecular replacement (MR) using different search models (Asojo et al., 2005, Asojo, 2011) with PHASER (Storoni et al., 2004, McCoy et al., 2005). Using Na-ASP-2 as the search model, a solution was obtained and the model was improved through automatic model building with ARP/wARP (Morris et al., 2003, Morris et al., 2004) followed by manual model building cycles using the programme Coot (Emsley et al., 2010) and structure refinement with REFMAC5 (Murshudov et al., 2011) within the CCP4 package (Winn et al., 2011) and Phenix (Terwilliger et al., 2008, Adams et al., 2010). The resulting model was comprised of amino acid residues, glycans, and water molecules. No electron density was observed that could be modelled as any lipids usurped during recombinant protein production. Ribbon diagram and model figures were generated using PyMOL (www.pymol.org). Details of the quality of the structure as well as data collection are shown in Table 1. The atomic coordinate and structure factors have been deposited in the protein databank (www.rcsb.org) under accession number 5WEE.

**Table 1:** Data collection and refinement statistics for *Heligmosomoides polygyrus* Venom Allergen-like Protein-4 (HpVAL-4).

Data collection	HpVAL-4
Wavelength	0.15418 nm
Resolution range (Å)	40.88–1.99 (2.061–1.99)
Space group	*P*_1_
Unit cell	*a* = 49.5596 Å *b* = 61.5645 Å *c* = 74.8188 Å *α* = 111.595° *β* = 90.14° *γ* = 113.475^o^
Total reflections	351,786 (23932)
Unique reflections	48,534 (4696)
Multiplicity	7.2 (7.3)
Completeness (%)	95.36 (92.93)
Mean I/sigma (I)	18 (11.3)
Wilson B-factor	16.45
R-merge	0.139 (0.700)
R-meas	0.156 (0.762)
R-pim	0.084 (0.421)
CC_1/2_	0.996 (0.961)
Reflections used in refinement	48,531 (4696)
Reflections used for R-free	2462 (225)
R-work	0.1748 (0.1824)
R-free	0.2231 (0.2702)
Number of non-hydrogen atoms	6464
Macromolecules	5921
Ligands	180
Solvent	363
Protein residues	749
RMS (bonds)	0.009 Å
RMS (angles)	1.39^o^
Ramachandran favoured (%)	97.71
Ramachandran allowed (%)	2.29
Ramachandran outliers (%)	0.00
Rotamer outliers (%)	0.15
Clashscore	4.05
Average B-factor	17.84
Macromolecules	17.50
Ligands	25.31
Solvent	19.72

Statistics for the highest resolution shell are shown in parentheses.

RMSD, root-mean-square deviation; CC, correlation coefficient.

### 2.6 Size exclusion chromatography and multi-angle light scattering (SEC-MALS)

SEC-MALS experiments were performed by loading ∼10 μg of protein sample onto a Phenomenex Yarra 3 µm SEC-2000 column (Phenomenex, Torrance, CA, USA) at a flow-rate of 0.5 ml/min using an Agilent 1260 Infinity series HPLC. The mobile phase was PBS buffer at pH 7.4. The elution was detected with a UV detector (Agilent, USA a miniDAWN triple-angle light-scattering detector (Wyatt Technology, USA) and with an Optilab rEX differential refractometer (Wyatt Technology) connected in series. The isotropic scatterer for detector normalisation was bovine serum albumin. Since the light scattered by a protein is directly proportional to its weight-average molecular mass and concentration, molecular masses were calculated from the light-scattering and interferometric refractometer data using ASTRA 6.1 software.

### 2.7 In vivo sterol export from mutant yeast cells

Acetylation and export of sterols into the culture supernatant was examined as previously described (Tiwari et al., 2007). Heme (*hem1*Δ) -deficient yeast cells were cultivated in presence of Cholesterol/Tween 80 containing media and labelled with 0.025 µCi/ml [^14^C] cholesterol (American Radiolabeled Chemicals Inc, St. Louis, MO, USA). Cells were harvested by centrifugation, washed twice with synthetic complete (SC) media, diluted to an O.D._600_ of 1 into fresh SC media containing non-radiolabeled cholesterol and grown overnight. Cells were centrifuged and lipids were extracted from the cell pellet and the culture supernatant using chloroform/methanol (v/v 1:1). Samples were dried and separated by thin-layer chromatography (TLC) using silica gel 60 plates (Merck, Darmstadt, Germany) using the solvent system, petroleum ether/diethyl ether/acetic acid (70:30:2; per vol.). Radiolabeled lipids on the TLC were quantified by scanning with a Berthold Tracemaster 40 Automatic TLC-Linear Analyzer (Berthold Technologies, Bad Wildbad, Germany). TLC plates were then exposed to phosphorimager screens and radiolabeled lipids were visualised using a phosphorimager (Bio-Rad Laboratories, Hercules, CA, USA).

### 2.8 In vitro sterol binding

In vitro sterol binding was assessed using a previously described radioligand-binding assay (Im et al., 2005, Choudhary and Schneiter, 2012, Darwiche and Schneiter, 2017)*.* Briefly, 100 pmol of purified protein in binding buffer (20 mM Tris, pH 7.5, 30 mM NaCl, 0.05% Triton X-100) were incubated with 0–400 pmol of [^3^H]-cholesterol (American Radiolabeled Chemicals Inc., St Louis, Missouri, USA) for 1 h at 30 °C. The protein was adsorbed to Q-Sepharose beads (GE healthcare) to remove unbound ligand, the beads were washed, and the radioligand was quantified by scintillation counting. For competition assays, 400 pmol of unlabeled cholesterol were included in the binding reaction, together with the indicated concentrations of [^3^H]-cholesterol. To determine non-specific binding, the ion exchange beads were incubated in the absence of added protein. At least two independent experiments were performed under each experimental condition and data are reported as the mean ± S.D. Calculation of the *K_d_* value and curve fitting were performed using the statistical software Prism (GraphPad, La Jolla, CA, USA).

## 3 Results

### 3.1 Recombinant HpVAL-4

**Fig. 1:**
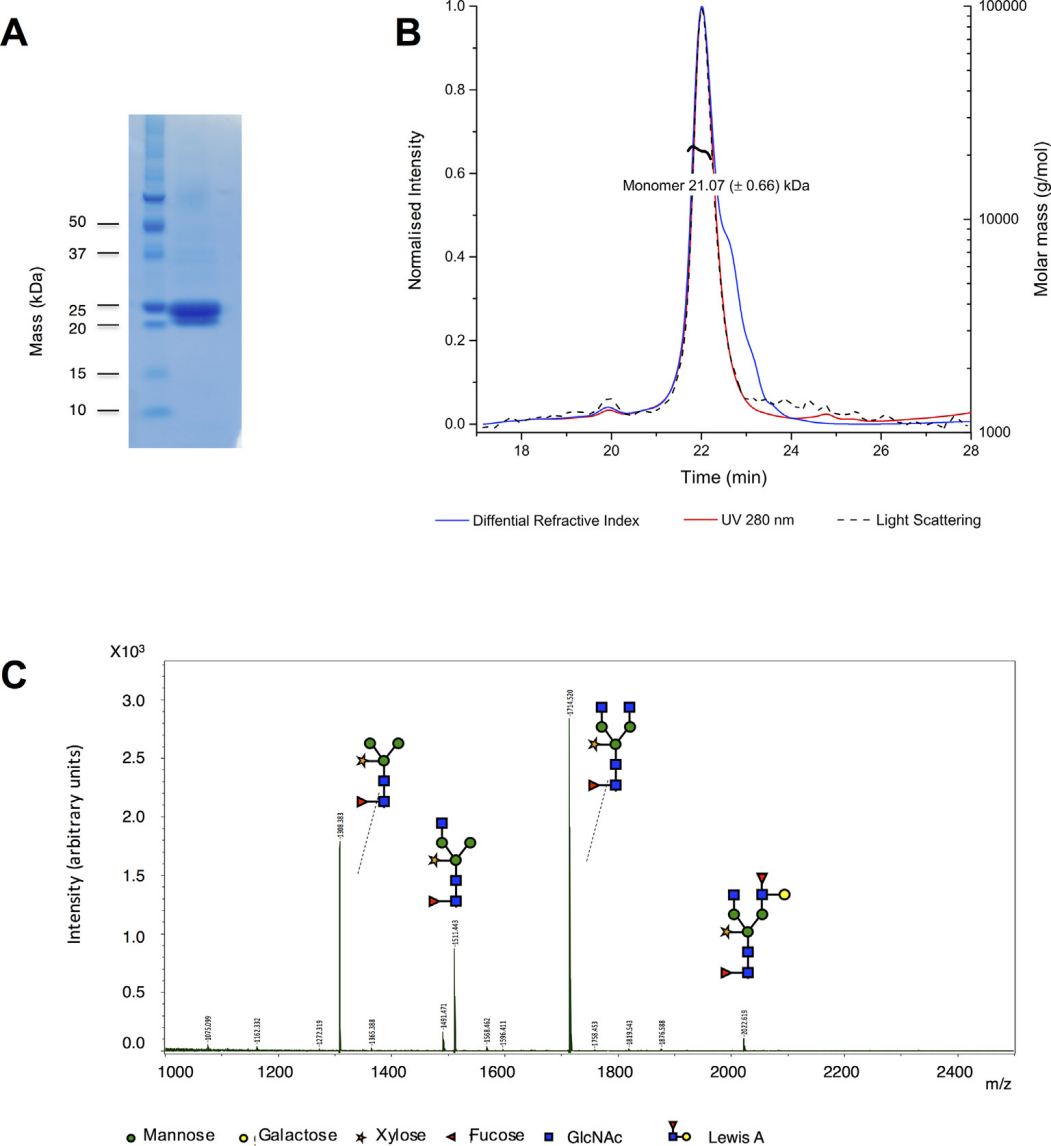
Protein purity and characterisation of *Heligmosomoides polygyrus* Venom Allergen-like Protein-4 (HpVAL-4). (A) Coomasie-stained SDS gel reveals the purity of recombinant HpVAL-4 and its monomeric mass of ∼22 kDa. (B) Size exclusion chromatography multi-angle light scattering analysis reveals that HpVAL-4 is an ∼21 kDa monomer in solution. (C) N-glycan composition of plant-produced HpVAL-4.

Expression of HpVAL-4 in our plant-based expression system resulted in a yield of 0.5–1.0 mg of pure recombinant protein per plant (3–4 g of leaf material). Recombinant HpVAL-4 was shown to be ∼95% pure by a Coomassie stained SDS–PAGE gel (Fig. 1A). The oligomeric state of recombinant HpVAL-4 in solution was determined by measuring the absolute molecular mass by SEC-MALS. The protein gave a single peak on the sizing column (Fig. 1B) with a molecular mass of 21.07 ± 0.66 kDa, consistent with its theoretical molecular mass of 21.7 kDa, indicating that HpVAL-4 forms a monomer in solution. It had been previously speculated that dimerisation was important for the functions of SCP/TAPS proteins (Asojo et al., 2005, Gibbs et al., 2008). Our ongoing studies reveal that while some SCP/TAPS proteins such as MpPR-1i and SmVAL-4 form monomers in solution (Kelleher et al., 2015, Baroni et al., 2017), others including GAPR-1, Na-ASP-2 and GLIPR-1 form dimers in solution (Asojo et al., 2005, Gibbs et al., 2008).

The glycosylation composition of HpVAL-4 was then assessed by MALDI-TOF MS analysis of released N-glycans. All N-glycan types found on the HpVAL-4 protein carry typical plant beta(1,2)-xylose and core alpha(1,3)-fucose residues (Fig. 1C). Furthermore, the majority of the N-glycans are biantennary with terminal GlcNAc residues (GnGnXF^3^), but also paucimannosidic N-glycans (MMXF^3^) and N-glycans with one terminal GlcNAc residue were detected (MGnXF^3^ or GnMXF^3^). These latter structures likely arise from apoplastic β-hexosaminidase activity (Shin et al., 2017). Altogether, our results demonstrate that plants are well suited as an expression platform for CAP proteins from helminths.

### 3.2 Crystal structure of HpVAL-4

**Fig. 2:**
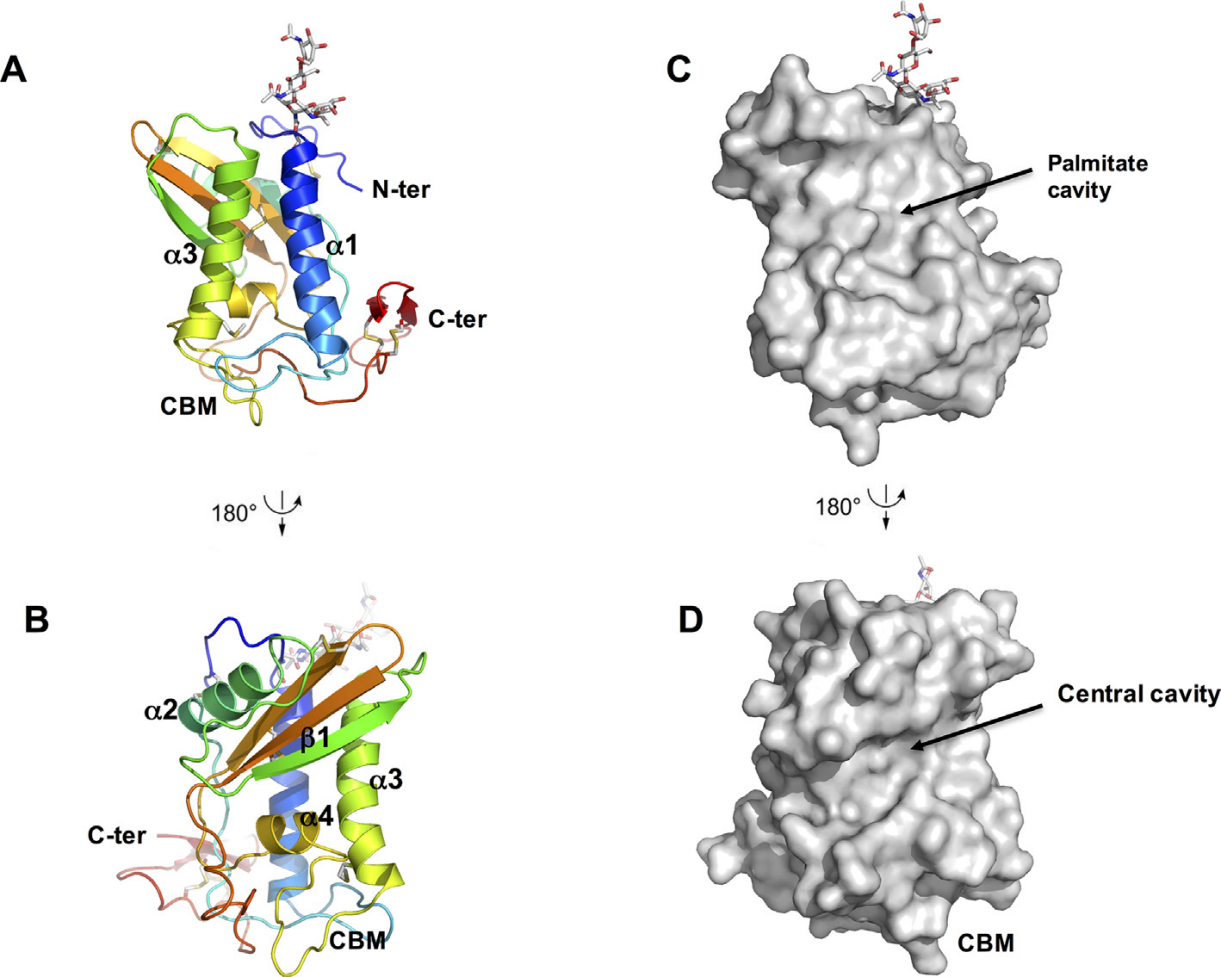
Crystal structure of *Heligmosomoides polygyrus* Venom Allergen-like Protein-4 (HpVAL-4). (A) Cartoon of a monomer of HpVAL-4 rainbow colours from amino (blue) to carboxyl terminus (red). The two longest helices α1 and α3 that form the palmitate cavity and the caveolin binding motif loop are indicated, while glycans and disulfide bridges are shown in stick form (coloured by elements: blue for N, white for C, red for O, and yellow for S). (B) Rotation (180 degrees) of the monomer allows better visualisation of the strand (β1) and helix (α4) that form the central cavity. (C, D) Surface representations of the views for A and B, respectively.

Each HpVAL-4 monomer folds as an alpha–beta–alpha sandwich, in which a beta sheet is sandwiched between two helical/loop regions (Fig. 2A). This classic SCP/TAPs motif is flanked by N-terminal and C-terminal extensions that are stabilised by disulfide bonds. While the refined model has four monomers of HpVAL-4 in the asymmetric unit (Supplementary Figs. S1 and S2), this tetramer is likely an artefact of crystallisation as SEC-MALS analysis indicates that HpVAL-4 is a monomer in solution. The monomers are very similar with root mean square deviation (rmsd) ranging between 0.075 Å and 0.176 Å for alignment of main chain atoms. The most variable regions between the monomers are loop regions, except the C-terminal extension that is virtually identical (Fig. 2B). The predicted N-linked glycosylation site Asn12 is glycosylated for all four monomers (Fig. 2A and B). The surface plot of HpVAL-4 reveals a large central CAP cavity of 1732.64 Å^3^, which is bordered by the first beta strand (β1) and the third and fourth alpha helices (α2,α4), and opens into a C-terminal loop (Fig. 2). This cavity is comparable in size to previously reported SCP/TAPs protein structures (Asojo et al., 2005, Asojo et al., 2011, Gibbs et al., 2008, Wang et al., 2010a, Asojo, 2011, Ma et al., 2011, Kelleher et al., 2015). Additionally, within the crystallographic tetramer there are no possible dimers that have packing similar to either the two-CAP Na-ASP-1 or the dimer in Pry1 that connect both central CAP cavities (Asojo, 2011, Darwiche et al., 2016).

### 3.3 HpVAL-4 rescues the cholesterol export defect in pry1Δ pry2Δ mutant cells and binds sterol in vitro

**Fig. 3:**
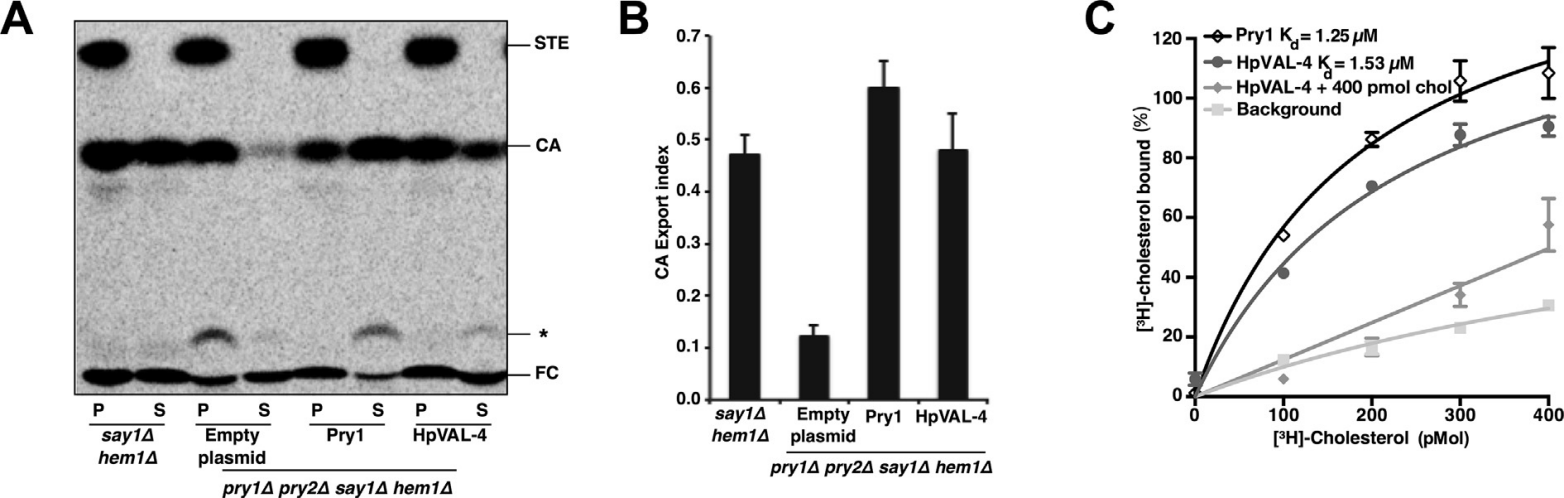
*Heligmosomoides polygyrus* Venom Allergen-like Protein-4 (HpVAL-4) exports and binds cholesterol in vivo and in vitro. (A) Expression of HpVAL-4 complements the sterol export defect of yeast cells lacking their endogenous cysteine-rich secretory proteins, antigen 5, and pathogenesis-related 1 (CAP) proteins (Pry1 and Pry2). Heme-deficient cells of the indicated genotype containing either an empty plasmid or a plasmid with Pry1 or HpVAL-4 were radiolabeled with [^14^C]cholesterol overnight, washed and diluted in fresh media to allow for export of cholesterol and cholesteryl acetate. Lipids were extracted from the cell pellet (P) and the culture supernatant (S), and separated by thin layer chromatography. The positions of free cholesterol (FC), cholesteryl acetate (CA) and steryl esters (STE) are indicated. The star marks the position of an unidentified cholesterol derivative. (B) Quantification of the export of cholesteryl acetate in yeast cells lacking their endogenous CAP proteins when complemented with empty plasmid, Pry1 or HpVAL-4. The export index indicates the relative percentages of cholesteryl acetate that is exported by the cells (ratio between the extracellular cholesteryl acetate and the sum of intra- and extra-cellular cholesteryl acetate). Data represent mean ± S.D. of two independent experiments. (C) HpVAL-4 binds cholesterol in vitro. Purified HpVAL-4 protein (100 pmol) was incubated with the indicated concentration of [^3^H]-cholesterol in the presence (HpVAL-4 + 400 pmol cholesterol) or absence of unlabeled competitor ligand (HpVAL-4). The previously determined Pry1 activity is also shown.

To test whether expression of HpVAL-4 in *pry1*Δ *pry2*Δ mutant cells rescued the defect in cholesterol export, heme-deficient cells containing either an empty plasmid or a plasmid with HpVAL-4 were radiolabeled with [^14^C]cholesterol overnight, washed and diluted in fresh media to allow for export of cholesterol and cholesteryl acetate. Lipids were extracted from the cell pellet (P) and the culture supernatant (S), and separated by thin layer chromatography (Fig. 3A). The levels of free cholesterol and cholesteryl acetate were quantified by radio scanning and the relative percentages of cholesteryl acetate exported by the cells were plotted as the export index which is the ratio between the extracellular cholesteryl acetate and the sum of intra- and extra-cellular cholesteryl acetate (Fig. 3B). Cells complemented with HpVAL-4 exported cholesteryl acetate into the culture supernatant at levels comparable to wild-type cells and Pry1 indicating that HpVAL-4 is an effective exporter.

HpVAL-4 was determined to bind cholesterol in vitro, by a direct binding assay in which increasing concentrations of [^3^H]-cholesterol (0–400 pmol) were incubated with 100 pmol of HpVAL-4 proteins. The protein was separated from unbound ligand by adsorption to an anion-exchange matrix and bound radioligand was quantified by scintillation counting. HpVAL-4 binds cholesterol in vitro with a saturable *k_d_* of 1.53 μM, which is comparable to that of Pry1 (*k_d_* of 1.25 μM) (Darwiche et al., 2016). Furthermore, cholesterol binding is specific as addition of unlabeled cholesterol (400 pmol) competes with radioligand binding (Fig. 3C). These binding studies show that HpVAL-4 binds [^3^H]-cholesterol in a concentration-dependent manner and that binding of the radiolabeled ligand can be competed with by incubation with unlabeled cholesterol (Fig. 3C).

## 4 Discussion

Using structural similarity in PDBeFold (http://www.ebi.ac.uk/msd-srv/ssm/), a three-dimensional (3-D) structural alignment that takes both the alignment length and rmsd into account, structures that were most similar to HpVAL-4 were identified as CAP proteins that have less than 30% sequence identity with HpVAL-4. The most similar structure to HpVAL-4 is that of Na-ASP-2, (Asojo et al., 2005, Mason et al., 2014) followed by human hookworm platelet inhibitor, HPI (Ma et al., 2015), *Ancylostoma*-secreted protein, Ac-ASP-7 (Osman et al., 2012), *Schistosoma mansoni* venom allergen-like protein 4, SmVAL4, (Kelleher et al., 2015), tablysin-15 (Ma et al., 2011), pry1 (Darwiche et al., 2016), human GAPR-1 (van Galen et al., 2012) and P14A from tomato (Fernandez et al., 1997). HpVAL-4 shares a conserved C-terminal extension with Na-ASP-2 that has two strands, which are stabilised by two disulfide bonds. This C-terminal extension is more varied in HPI, SmVAL4 and Ac-ASP-7. The flexible N-terminal loops of both proteins also have a conserved disulfide bond with alpha helix 2.

**Fig. 4:**
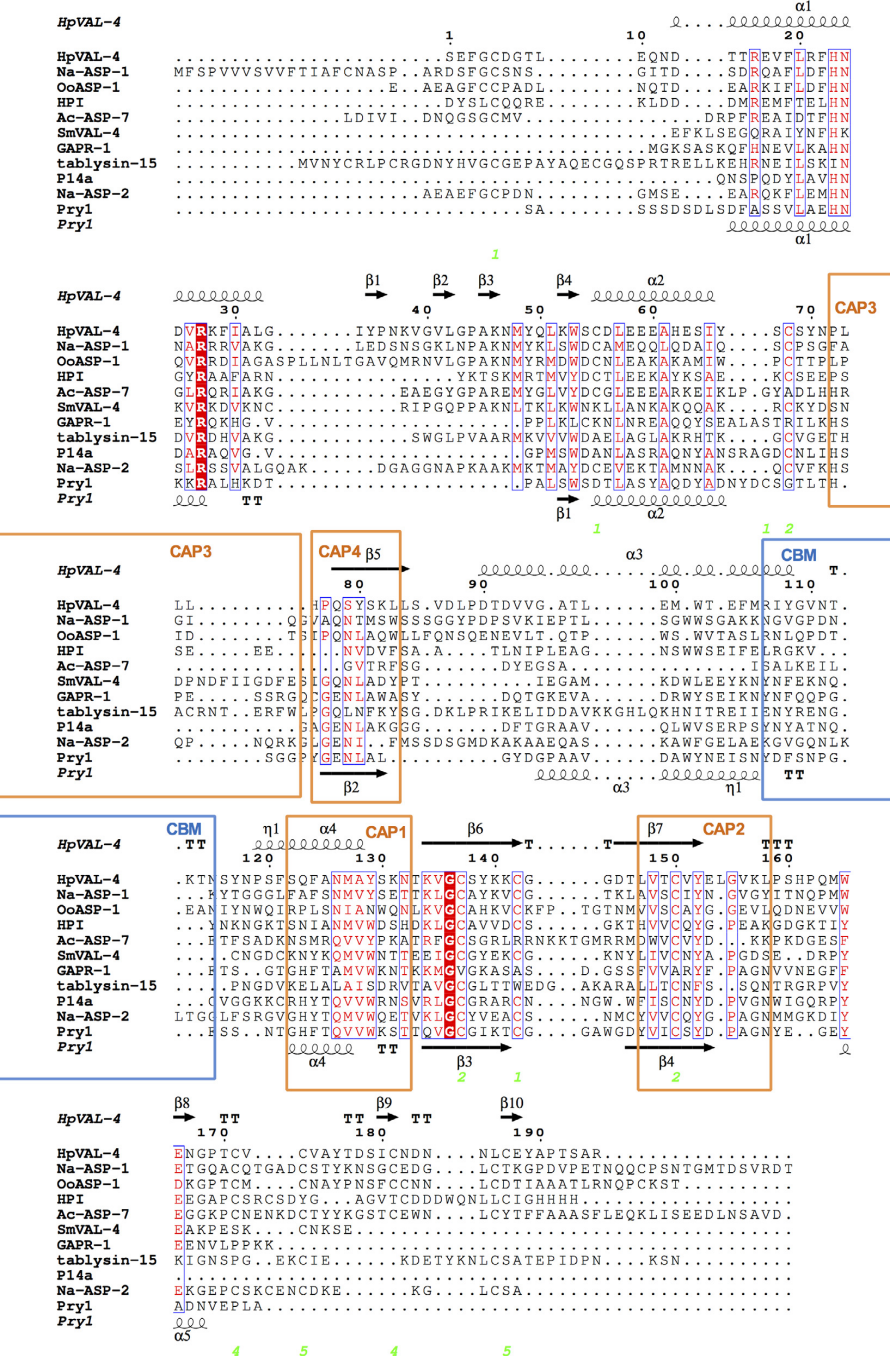
Comparison of *Heligmosomoides polygyrus* Venom Allergen-like Protein-4 (HpVAL-4) with selected members of its superfamily. The sequences were aligned with clustalW2 and the secondary structural features were illustrated with the coordinates of HpVAL-4 and Pry1 using ESPript (Gouet et al., 2003). The different secondary structure elements shown are alpha helices (α), 3_10_-helices (η), beta strands (β), and beta turns (TT). Identical residues are shown in red shading, and conserved residues are in red text. The locations of the cysteine residues involved in disulfide bonds are numbered in green. The location of the caveolin binding motif loop is shown in blue bars and the signature cysteine-rich secretory protein (CRISP) motifs are identified with orange bars. The representative structures are Na-ASP-2 (Asojo et al., 2005), Pry1 (Darwiche et al., 2016), Na-ASP-1 (Asojo, 2011), tablysin-15 (Ma et al., 2011), Golgi-Associated plant Pathogenesis Related protein-1, GAPR-1 (van Galen et al., 2012), *Ostertagia ostertagi* activation-associated secreted protein-1, OoASP-1 (Borloo et al., 2013), *Ancylostoma caninum* Ancylostoma secreted protein-7, Ac-ASP-7 (Osman et al., 2012), *Schistosoma mansoni* Venom Allergen-like Protein-4, SmVAL4, (Kelleher et al., 2015), and *Solanum lycopersicum* pathogenesis-related protein, P14A (Fernandez et al., 1997).

Only two proteins with greater than 30% sequence identity with HpVAL-4 have reported crystal structures, *Necator americanus* ASP-1 (Asojo, 2011) and *Ostertagia ostertagi* ASP-1 (Borloo et al., 2013). Neither was a top hit based on PDBeFold analysis due to the variations in the loop regions that make up over 45% of the topology of CAP proteins. Interestingly, reverse template alignment using ProFunc (Laskowski et al., 2005, Laskowski, 2017) reveals that both structures are certain matches (E-value < 1.00E−06) and while there are indeed insertions and gaps in loop regions, the topology of many of the helices and strands are conserved (Supplementary Figs. S3 and S4). As previously mentioned, the loop regions are flexible and varied for SCP/TAPS protein structures and make it difficult to model the structures. Furthermore, even among those that share structural similarity with HpVAL-4, the lengths of loops differ (Fig. 4).

Despite the difference in the orientation of its loop region, HpVAL-4 has a central exposed cavity similar to previously reported SCP/TAPS protein structures (Asojo et al., 2005, Asojo et al., 2011, Gibbs et al., 2008, Asojo, 2011). Since HpVAL-4 lacks the histidines that bind divalent cations, it is unable to coordinate Zn^2+^ for the proposed heparin-sulphate-dependent mechanisms of inflammatory modulation by the cobra CRISP natrin (Wang et al., 2010a). The sequence of amino acid residues in the CAP cavity of HpVAL-4 is more similar to that of the N-terminal CAP domain of Na-ASP-1 (Fig. 4, Supplementary Fig. S4). Comparison of the CAP cavity suggests that HpVAL-4 and the N-terminal CAP domain of Na-ASP-1 may present a different motif for hookworms and more studies are needed to identify the biological relevance of the conserved residues.

**Fig. 5:**
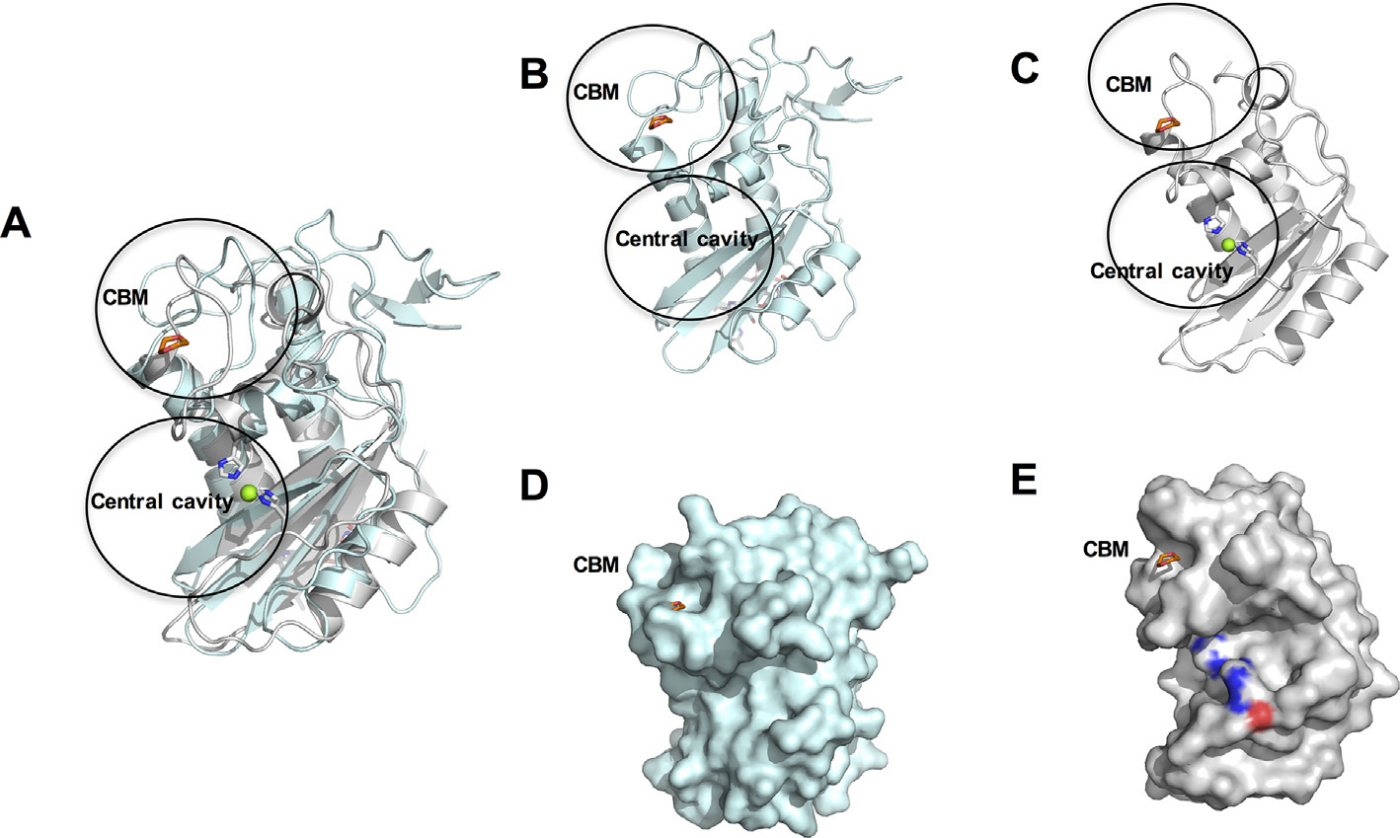
Comparison of *Heligmosomoides polygyrus* Venom Allergen-like Protein-4 (HpVAL-4) with pathogen-related yeast protein 1 (Pry1). (A) The superposed ribbon structure of HpVAL-4 (cyan) and Pry1 (grey) reveals the conformational flexibility of the caveolin binding motif which contains the 1,2-dioxane from the Pry1 structure (shown in red). The central histidines that coordinate cations in Pry1 are coloured by elements with blue for N, white for C, red for O. Mg^2+^ is shown as a green sphere. Ribbon diagrams of the same view of (B) HpVAL-4 and (C) Pry1. The sizes of the cavities are evident from the surface plot of the same view of (D) HpVAL-4 and (E) Pry1.

It was previously shown that *Saccharomyces cerevisiae* CAP proteins are required for cholesteryl acetate transport (Choudhary and Schneiter, 2012, Choudhary et al., 2014) and we now report that HpVAL-4 rescues the sterol-binding function of yeast that lacked the endogenous CAP proteins Pry1 and Pry2, and that recombinant HpVAL-4 binds sterol in vitro (Fig. 3). While HpVAL-4 has less than 30% sequence similarity to Pry1 and Pry2, it also shares limited structural similarity with Pry1 (Fig. 4). It has been shown that a caveolin-binding motif (CBM), which is located in a flexible loop region connecting helices α3 and α4 (Fig. 2, Fig. 4), that has several polar amino acid residues capable of interacting with lipids, is important for both in vivo and in vitro sterol binding by Pry1 (Choudhary et al., 2014). The amino acid sequence of the CBM is characterised by the presence of conserved aromatic amino acids, which are required for the in vivo export and the in vitro binding of sterols in SCP/TAPS proteins. The CBM loop of HpVAL-4 has a different conformation from Pry1 but creates a cavity that is large enough to accommodate dioxane, as was observed in the structure of Pry1 (Fig. 5). Interestingly, the insertion of a string of glutamic acid residues makes the cavity less hydrophobic than observed for Pry1 (Fig. 4). Our analysis shows that HpVAL-4 has comparable in vitro cholesterol binding ability to Pry1 (Fig. 3C).

**Fig. 6:**
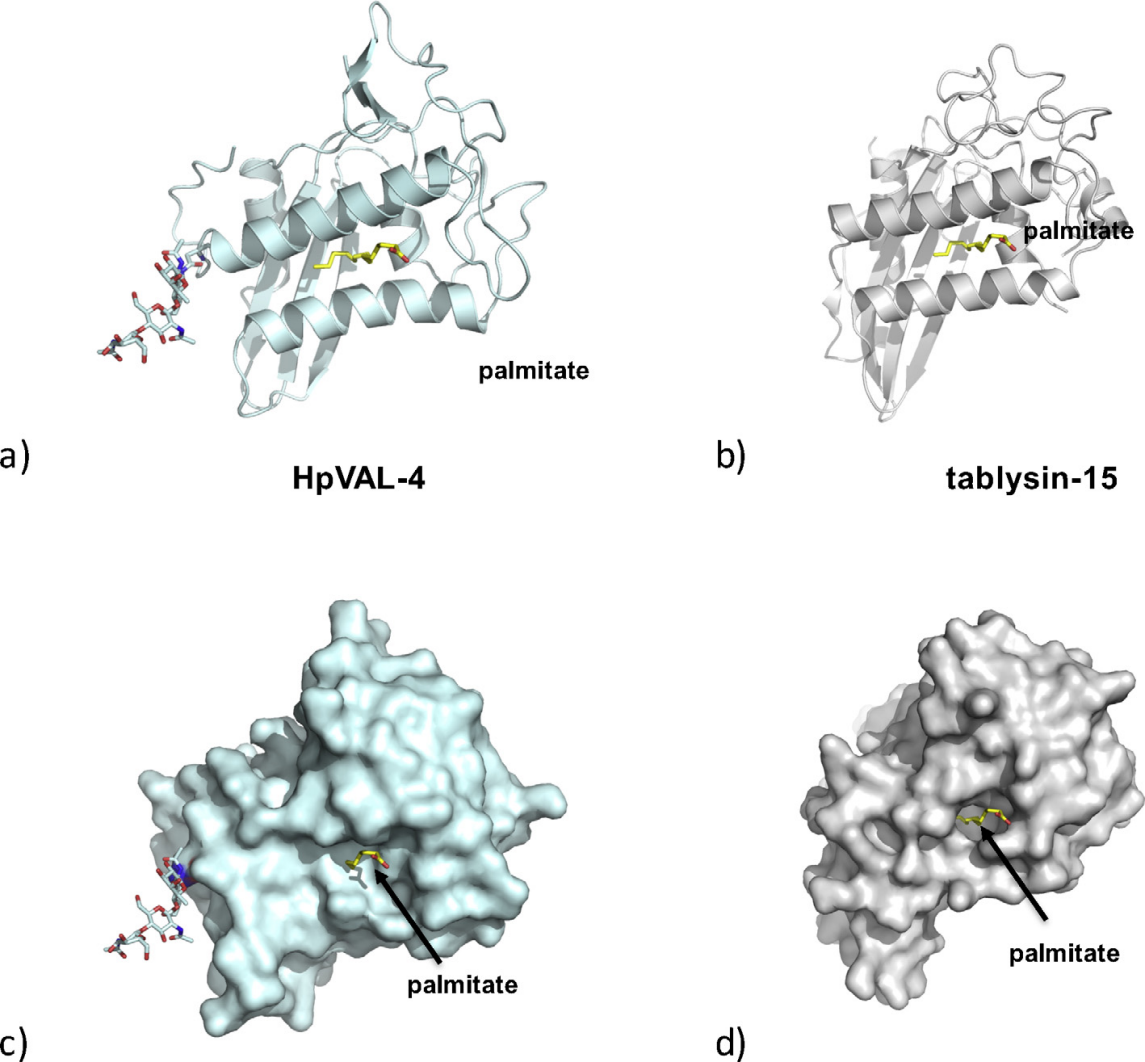
The palmitate binding cavity. (A) Ribbon diagram of the putative palmitate-binding cavity of *Heligmosomoides polygyrus* Venom Allergen-like Protein-4 (HpVAL-4) based on (B) the palmitate binding of tablysin-15. Surface representations of same view of the palmitate binding cavity of (C) HpVAL-4 and (D) tablysin-15, with palmitate shown as yellow sticks and the glycosylation site on HpVAL-4 as other coloured sticks.

A second distinct and independent lipid-binding function of SCP/TAPS proteins is the palmitate binding observed in a cavity between two helices as observed in tablysin-15. This cavity was also shown to bind leukotriene (Xu et al., 2012). The locations of alpha helices 1 and 4 are conserved in SCP/TAPS proteins including HpVAL-4 (Fig. 2, Fig. 4). As previously indicated for other CAP proteins, the amino acid residues in the palmitate binding cavity are poorly conserved, however there is sufficient space between the equivalent helices to facilitate palmitate binding (Fig. 2, Fig. 6). These analyses reveal that HpVAL-4 is structurally able to bind palmitate, as was observed for tablysin-15. This suggests that HpVAL4 may be able to bind other fatty acids and fatty acid-derived products such as the immunologically relevant prostaglandins and leukotrienes. Such a role in immune modulation, rather than lipid transport within the *H. polygyrus* organism, is also indicated by its expression across all mammalian stages of the parasite, and its prominent secretion by both tissue-stage larvae and luminal-dwelling adults of the species (Hewitson et al., 2013). Further studies will shed light on whether HpVAL-4 indeed has such an immunomodulatory function.

Hp-VAL-4 produced in our plant expression system is a glycosylated protein that readily crystallised from high concentrations of PEG. HpVAL-4 is a monomer in solution and retains a large open palmitate-binding cavity, making it capable of binding this and other lipids. Additionally, the presence of a large CBM explains the ability of HpVAL-4 to export sterol in vivo. HpVAL-4 has a large central cavity that lacks the prototypical CAP cavity tetrad, which means it will be incapable of binding divalent cations. The amino acid residues in the CAP cavity of HpVAL-4 are more similar to those in the amino terminal CAP domain of the two CAP Na-ASP-1. Studies are underway to determine endogenous binding partners of HpVAL-4 and other SCP/TAP proteins from parasites.

## Appendix A Supplementary data

Supplementary Figs. 1–4 Supplementary Fig. S1. *Heligmosomoides polygyrus* Venom Allergen-like Protein-4 (HpVAL-4) crystallographic tetramer packing shows no appreciable interaction between monomers. Supplementary Fig. S2. Superposition of the *Heligmosomoides polygyrus* Venom Allergen-like Protein-4 (HpVAL-4) monomers reveals extensive structural similarity. Supplementary Fig. S3. The secondary structure alignment of *Heligmosomoides polygyrus* Venom Allergen-like Protein-4 (HpVAL-4) with *Ostertagia ostertagi* activation-associated secreted protein-1 (OoASP-1) reveals conserved secondary structure motifs and variations in loop regions. Supplementary Fig. S4. The secondary structure alignment of *Necator americanus Ancylostoma* secreted protein-1 (Na-ASP-1) reveals conserved secondary structure motifs and variations in loop regions.
