# Supplementary figures and images for: Heligmosomoides polygyrus Venom Allergen-like Protein-4 (HpVAL-4) is a sterol binding protein
Source: Int J Parasitol. 2018 Apr;48(5):359–69. doi: 10.1016/j.ijpara.2018.01.002 (PMC5893428; doi:10.1016/j.ijpara.2018.01.002)

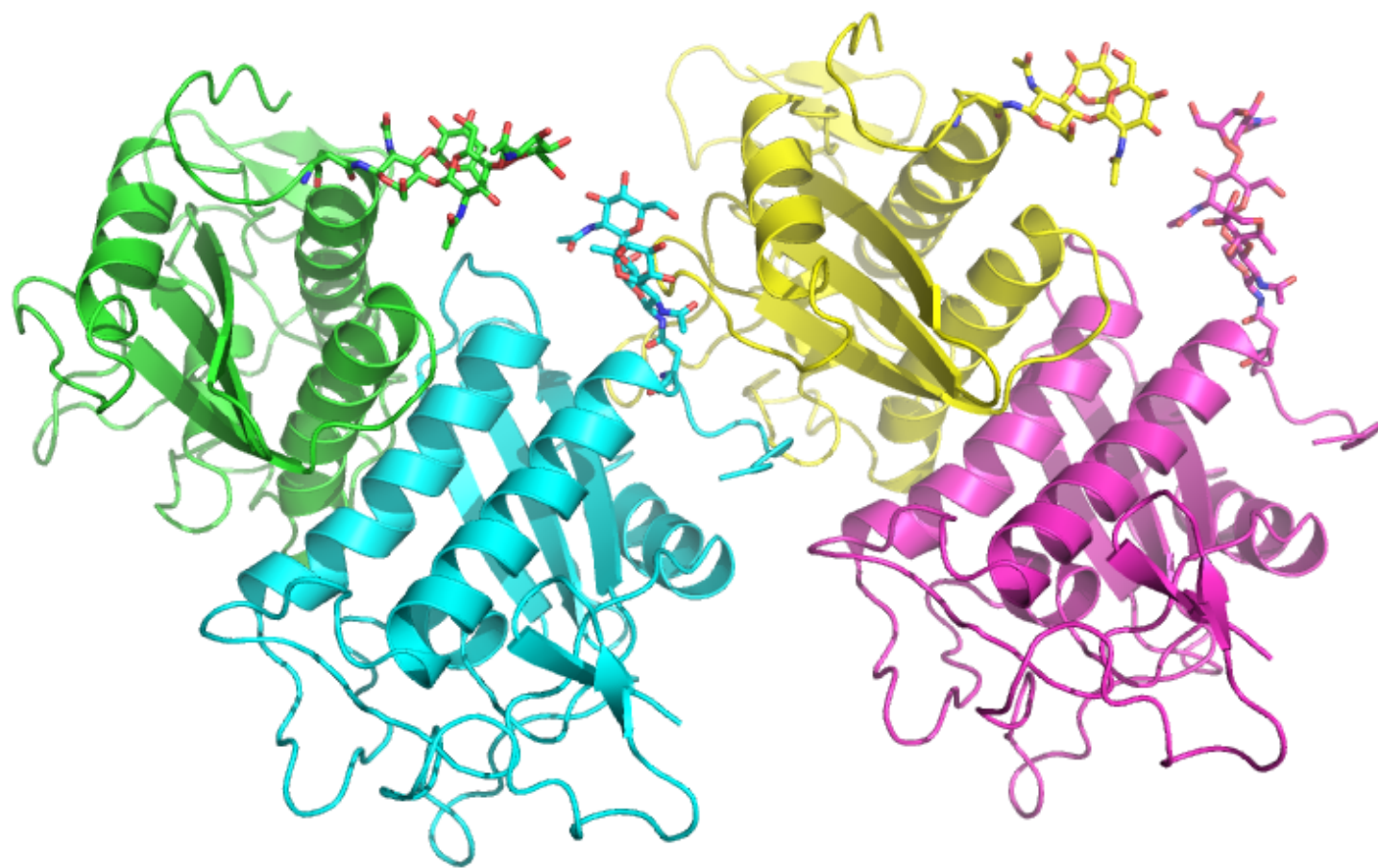

Supplementary Fig. S1.

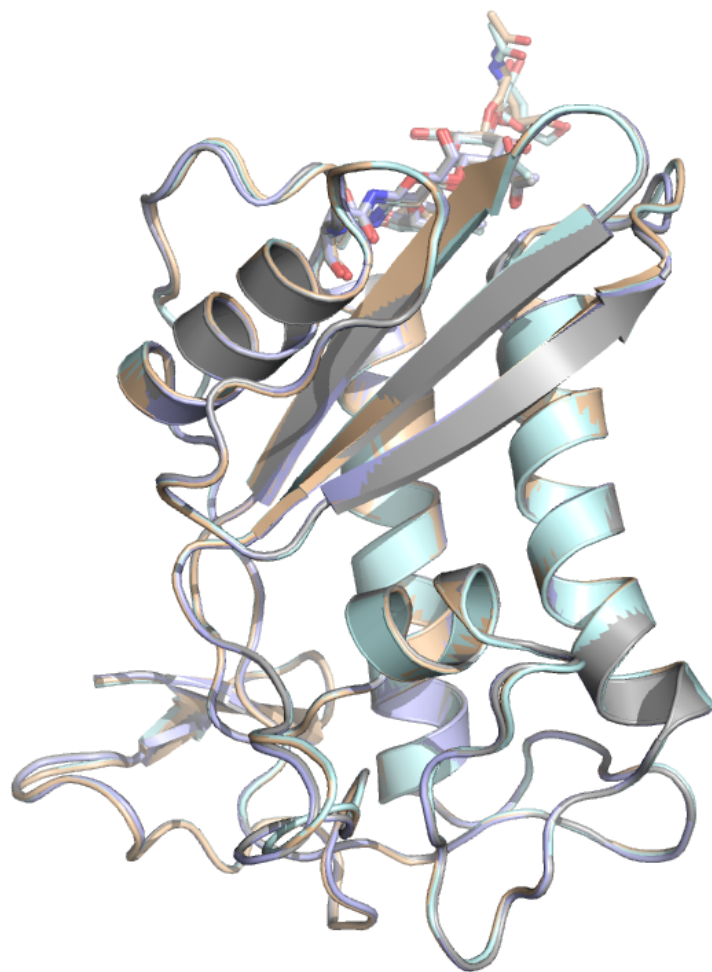

Supplementary Fig. S2.

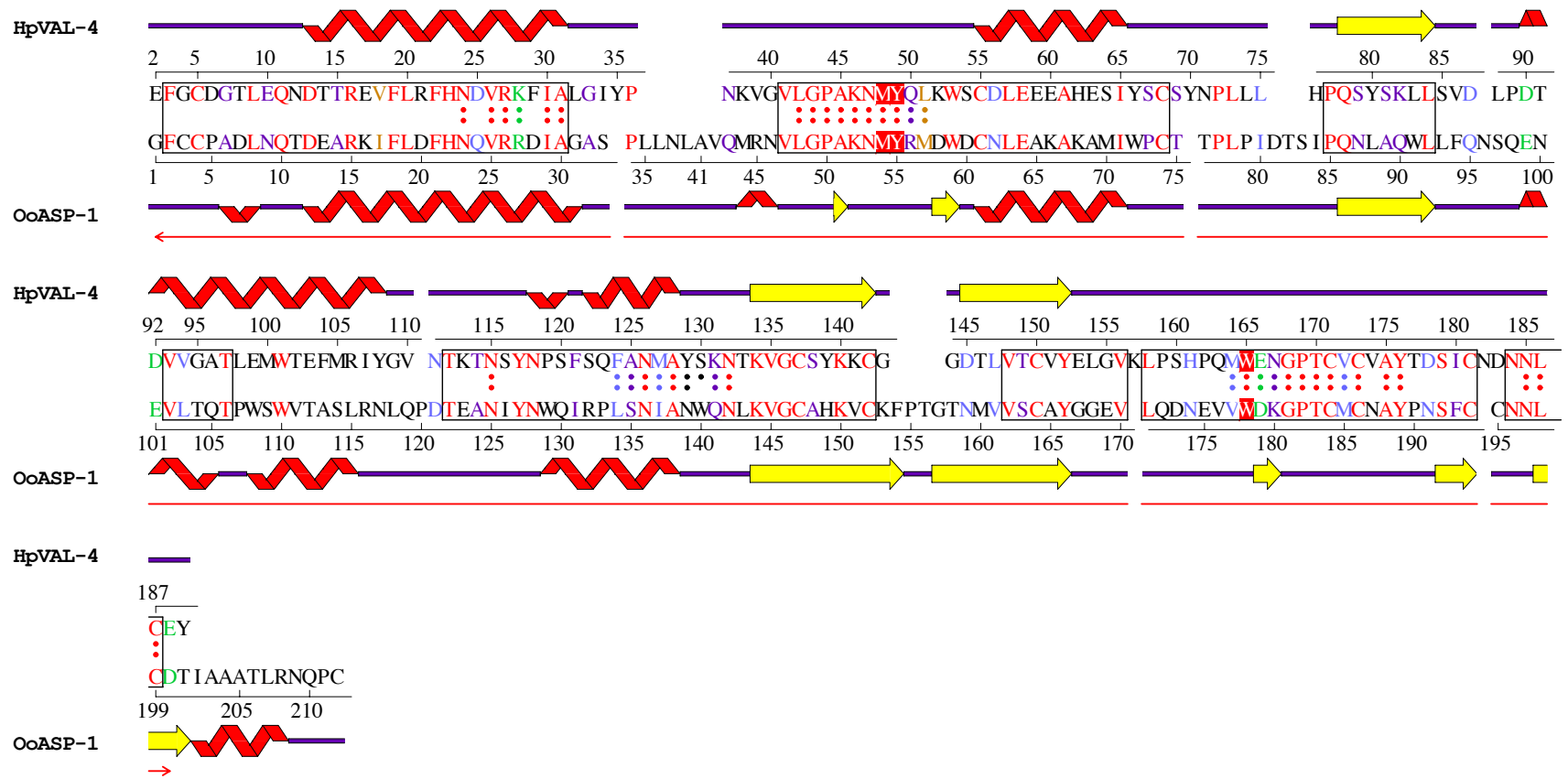

Supplementary Fig. S3.

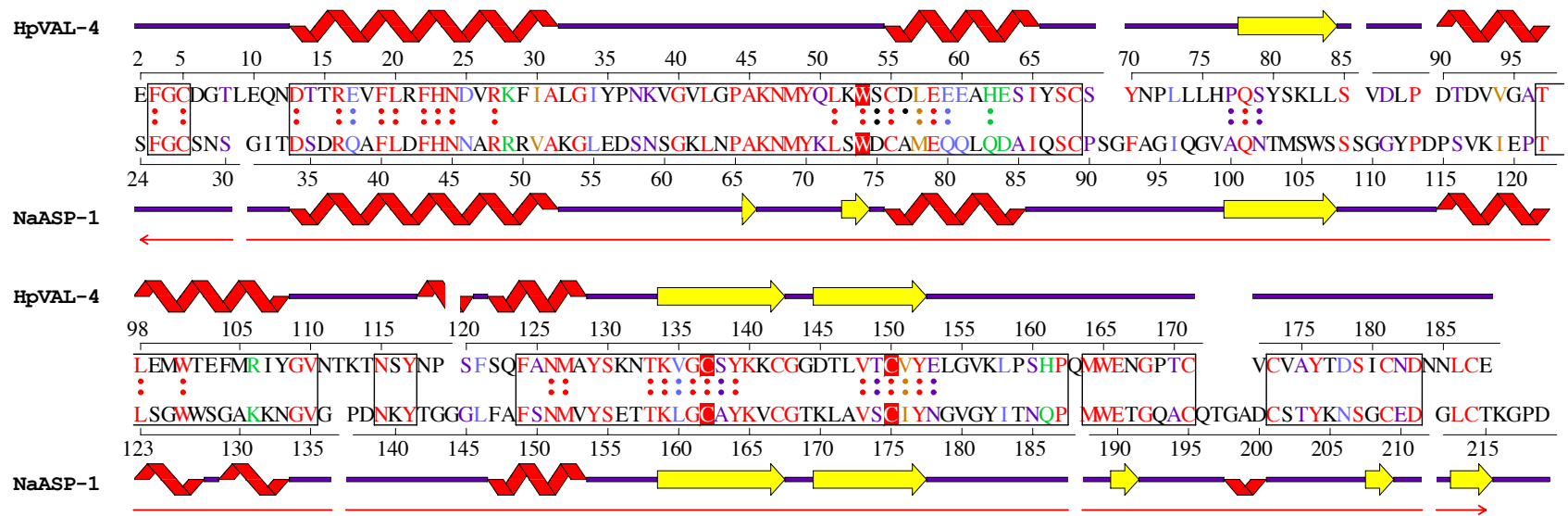

Supplementary Fig. S4.

Supplement: Supplementary Figs. 1–4 — Supplementary Fig. S1. Heligmosomoides polygyrus Venom Allergen-like Protein-4 (HpVAL-4) crystallographic tetramer packing shows no appreciable interaction between monomers. Supplementary Fig. S2. Superposition of the Heligmosomoides polygyrus Venom Allergen-like Protein-4 (HpVAL-4) monomers reveals extensive structural similarity. Supplementary Fig. S3. The secondary structure alignment of Heligmosomoides polygyrus Venom Allergen-like Protein-4 (HpVAL-4) with Ostertagia ostertagi activation-associated secreted protein-1 (OoASP-1) reveals conserved secondary structure motifs and variations in loop regions. Supplementary Fig. S4. The secondary structure alignment of Necator americanus Ancylostoma secreted protein-1 (Na-ASP-1) reveals conserved secondary structure motifs and variations in loop regions. [file mmc1.pdf]
